# Ovarian Hormones Regulate the Production of Adipocytes From Bone Marrow-Derived Cells

**DOI:** 10.3389/fendo.2018.00276

**Published:** 2018-05-28

**Authors:** Kathleen M. Gavin, Timothy M. Sullivan, Wendy M. Kohrt, Susan M. Majka, Dwight J. Klemm

**Affiliations:** ^1^Division of Geriatric Medicine, School of Medicine, University of Colorado Anschutz Medical Campus, Aurora, CO, United States; ^2^Charles C. Gates Center for Regenerative Medicine and Stem Cell Biology, School of Medicine, University of Colorado Anschutz Medical Campus, Aurora, CO, United States; ^3^Geriatric Research, Education and Clinical Center, VA Eastern Colorado Heath Care System, Denver, CO, United States; ^4^Division of Pulmonary Sciences and Critical Care Medicine, School of Medicine, University of Colorado Anschutz Medical Campus, Aurora, CO, United States; ^5^Cardiovascular Pulmonary Research Laboratory, School of Medicine, University of Colorado Anschutz Medical Campus, Aurora, CO, United States; ^6^Division of Allergy, Pulmonary, and Critical Care Medicine, Department of Medicine, Vanderbilt University School of Medicine, Nashville, TN, United States

**Keywords:** adipocyte, estrogen receptor, bone marrow-derived cells, bone marrow transplant, ovarian hormones, myeloid cells

## Abstract

Sex differences in body fat distribution and menopause-associated shifts in regional adiposity suggest that sex hormones play an important role in regulating the differentiation and distribution of adipocytes, but the underlying mechanisms have not been fully explained. The aim of this study was to determine whether ovarian hormone status influences the production and distribution of adipocytes in adipose tissue arising from bone marrow-derived cells. Nine- to ten-week-old ovariectomized (OVX), surgery naïve (WT), and estrogen receptor alpha knockout (αERKO) mice underwent bone marrow transplantation from luciferase or green fluorescent protein expressing donors. A subset of OVX animals had estradiol (E_2_) added back. Eight-weeks posttransplant, whole body and gonadal fat BM-derived adipocyte production was highest in OVX and αERKO mice, which was attenuated in OVX mice by E_2_ add-back. All groups demonstrated the highest bone marrow derived adipocyte (BMDA) production in the gonadal adipose depot, a visceral fat depot in mice. Taken together, the loss of ovarian hormones increases the production of BMDAs. If translatable across species, production of BMDA may be a mechanism by which visceral adiposity increases in estrogen-deficient postmenopausal women.

## Introduction

Obesity is an ever-growing epidemic in the United States and around the world (1, 2). However, it is now clear that the disease risk conferred with obesity does not simply depend on overall adiposity, but on body fat distribution (3, 4). In fact, the cardiometabolic disease protection observed in women before the menopausal transition can be attributed, at least in part, to preferential accumulation of fat in peripheral adipose depots, primarily the hips and thighs (5). Notably, even without weight gain, peri- and postmenopausal women experience a shift in body fat distribution toward both subcutaneous and visceral abdominal adipose tissue depots (6, 7). Thus, sex hormones, and estrogen status, in particular, are hypothesized to play a mechanistic role in determining body fat distribution, although the specifics of this regulatory mechanism have yet to be completely described.

Depot-specific differences in gene expression and metabolic phenotype of adipocytes have been previously described (8–10). This phenotypic variation may be due to differences in the progenitor populations from which new adipocytes are developed (9, 11). Of particular interest is a subset of adipocytes in the white adipose tissue of mice that arise *de novo* from an unconventional progenitor source, bone marrow-derived cells (BMDCs). These bone marrow derived cells of the hematopoietic myeloid lineage traffic through the circulation to the adipose tissue and commit to the adipocyte lineage in mice (12–16) and humans (17, 18).

Mouse studies have shown that bone marrow-derived adipocytes (BMDA) appear in a sex- and age-specific manner, with the greatest accumulation in the adipose tissue of older female mice (15). Interestingly, the regions that display the greatest accumulation of BMDA are those typically associated with metabolic dysfunction, the epi/pericardial and gonadal adipose depots (15, 16, 18). Thus, we hypothesize that the suppression of gonadal hormone signaling regulates the development of BMDAs, resulting in their specific accumulation central adipose tissue depots.

Rodent models are often utilized in mechanistic studies of obesity. In mice, ovariectomy (OVX) is used as a model of the postmenopausal state, recapitulating the increase in body weight and, more importantly, the redistribution of body fat to central depots (19, 20) observed in postmenopausal women. Furthermore, estrogen receptor knockout models have convincingly demonstrated a role for estrogen in regulating fat mass (21–23). The observation that estrogen receptor alpha knockout (αERKO) mice have a greater fat mass than WT mice suggests that ERα plays a protective role against fat accumulation (21). In fact, Pedram et al. demonstrated that ERα plays a specific role in suppressing adipogenesis (24).

Based upon our previous findings that BMDA accumulation increases with age and studies demonstrating both OVX and αERKO increase adiposity we utilized surgical OVX with or without the administration of exogenous 17β-estradiol (E_2_) combined with lineage labeled bone marrow transplantation (BMT) models [e.g., green fluorescent protein (GFP) or luciferase] to determine the influence of gonadal hormone status on BMDA accumulation. We also utilized a whole-body αERKO mouse model to interrogate disruptions in E_2_ signaling through ERα as a mechanism by which ovarian hormones may regulate BMDA production. We hypothesized that OVX and αERKO would result in increased production of BMDAs in metabolically detrimental depots (e.g., gonadal), and that this increase would be attenuated by estrogen replacement.

## Materials and Methods

### Animals

All animal procedures were performed in an AAALAC-accredited facility in accordance with the Guide for the Care and Use of Laboratory Animals (25) and approved by the University of Colorado (CU) Denver Institutional Animal Care and Use Committee. Wild-type C57BL/6J mice from The Jackson Laboratory (#000664) were used in all experiments unless otherwise stated. If applicable, OVX was performed by The Jackson Laboratory at 6 weeks of age, and all animals, male and female, arrived at CU at 8 weeks of age. Additional transgenic animals purchased from The Jackson Laboratory included estrogen receptor alpha null (αERKO) mice (#004744), adiponectin promoter driven cre-recombinase (AdipoQ-cre) expressing mice (#010803), adipocyte protein 2 driven cre-recombinase (aP2-Cre) expressing mice (#005069), and mice expressing enhanced GFP driven by the human ubiquitin-C promoter (#004353). *LoxP*/stop/*loxP* (LSL) Luciferase mice were acquired from the National Cancer Institute’s mouse repository (stock #01XAC). *Cre*-recombinase and *loxP* expressing animals were bred in-house to create hemizygous cre and hemizygous lox expressing animals to use in future studies. Mice were maintained on a 12 h light, 12 h dark schedule at room temperature (22°C) with *ad libitum* access to water and standard rodent chow free of phytoestrogens, Envigo Teklad global soy protein-free extruded diet (#2920X), throughout all studies.

### Bone Marrow Transplantation

Bone marrow donors were euthanized by isoflurane inhalation and cervical dislocation. Fresh BM cells were harvested aseptically from the femurs and tibias using a 27-gauge needle/syringe and 2 ml phosphate-buffered saline. The cells were disaggregated by gentle pipetting several times and filtered to obtain the single cell suspension utilized in transplantation.

Male and female animals necessitating BMT for lineage tracing studies underwent transplantation after 1–2 weeks of acclimatization (at approximately 9–10 weeks of age). Recipient mice were irradiated with a 12 Gy total dose, split into 6 Gy doses, separated by 4 h, using an X-ray radiation source. Immediately following the second dose, recipients were injected *via* the retro-orbital venous plexus with 1 × 10^6^ BM cells suspended in 100 µl sterile saline.

The BM used for transplantation came from one of the following transgenic mouse strains: AdipoQ-cre X LSL-Luciferase, aP2-cre X LSL-Luciferase or Ubc-GFP donors. AdipoQ-cre and aP2-cre mice use the adipocyte-specific adiponectin (AdipoQ) or fatty acid binding protein 4 (aP2) gene promoters, respectively, to drive expression of cre-recombinase. The LSL-Luciferase mice carry a luciferase reporter gene downstream of a loxP-flanked stop codon. In cells expressing cre-recombinase, the stop codon is excised allowing expression of the luciferase reporter gene. Thus, BM from dual transgenic AdipoQ-cre x LSL-Luciferase or aP2cre x LSL-Luciferase mice has a luciferase reporter gene that is only expressed in mature adipocytes. When this BM is transplanted into WT mice, only mature adipocyte arising from the transplanted BM will express luciferase, identifying BMDAs. Ubc-GFP mice exhibit expression of GFP under control of the human ubiquitin-C promoter in all cell types throughout the body. Thus, all cells arising from BM transplanted from Ubc-GFP mice will be positive for GFP, including mature adipocytes of the BM lineage.

### Hormone Replacement in Female Mice

One to two weeks after BMT (at approximately 10–12 weeks of age, approximately 6 weeks after OVX), hormone replacement was initiated *via* implantation of subcutaneous pellets in those mice randomized to the E_2_ add-back group (Innovative Research of America, Estradiol: Cat. # SE-121, 0.05 mg/pellet, 60-day release) or a sham surgery control group. Pellets were implanted in the dorsal subcutaneous tissue of the mouse. One E_2_ pellet was introduced *via* 10-gauge trocar. Implants were expected to maintain circulating hormone levels in the physiological range with the doses previously utilized in other mouse studies of adipogenesis and E_2_ replacement after OVX (26).

### Whole-Body Luciferase Imaging

Whole-body luciferase activity was completed with the IVIS Imaging System 50. Animals were lightly anesthetized and injected with d-luciferin (120 mg/kg, 100 µl retro-orbital). Measurements were initiated 3 min after luciferin injection, and luminescence was integrated over 5 min. In some animals, *in vivo* imaging was repeated at 2, 4, 6, 8, and 10 weeks posttransplant.

At the end of the study, mice were euthanized by CO_2_ asphyxiation and cervical dislocation. Body weight of the mice was measured immediately before sacrifice (18 weeks of age, 8–10 weeks posttransplant). Gonadal, inguinal, and interscapular (mix of white and brown fat) adipose depots and if appropriate, lower hind limb skeletal muscle (combined gastrocnemius and soleus), liver, and lung, were recovered, weighed, and processed for appropriate assay.

### Staining and Flow Cytometry

Adipose tissue was minced into pieces of 1–3 mm. Tissue fragments were digested at 37°C for 1 h with gentle shaking in digestion buffer [Krebs–Ringer HEPES + 2.5 mM glucose + 2% fetal bovine serum (FBS) (Gemini Bio-products, #100–500) + 200 nM adenosine (Sigma Aldrich, #A4036) + 1 mg/ml collagenase (type VIII, Sigma Aldrich, C2139), pH 7.4] using 4 ml buffer/gram of fat. Samples were then passed through a 150 µm Celltrics filter (Sysmex Partec GmbH, Germany, #04-004-2324), and digestion stopped by the addition of one volume of wash buffer [Hanks Balanced Salt Solution (Mediatech, #21-022-CV) + 2% FBS + 200 nM adenosine, pH 7.4]. Adipocytes were separated from the stromal pellet *via* centrifugation at 300 *g* 10 min, and the wash step repeated to ensure sufficient separation and exclusion of digestion buffer. The adipocyte and stromal fractions were collected and prepared for appropriate experiments.

#### Adipocyte Preparation

Human TruStain FcX™ (Fc Receptor Blocking Solution, Cat. No. 422301; BioLegend, San Diego, CA, USA) was added to isolated adipocytes at 5µl/100 µl of cell suspension, and cells incubated at room temperature for 10 min. LipidTOX Deep Red Neutral Lipid Stain (Cat. No. H34477; Life Technologies, Thermo Fisher Scientific Inc., Waltham, MA, USA, 1:200 dilution) and Vybrant^®^ DyeCycle™ Violet stain (Invitrogen, Cat. # V35003, 1 µl/10^6^ cells) were added to the cell suspension and incubated protected from light at 37°C for 30 min. Cells were kept at 37°C until analysis. Cytosolic GFP expression was assessed in intact adipocytes (LipidTOX Red^POS^ events) containing a single nucleus (DyeCycle violet^POS^ events) by flow cytometry as detailed previously.

#### Stromal Cell Preparation

The stromal cell pellet was resuspended in eBioscience 1X Red Blood Cell Lysis Buffer (#00-4300-54) at room temperature for 5 min. Stromal cells were pelleted by centrifugation at 500 *g* 5 min and washed by resuspension in 5 ml wash buffer (HBSS with 1% FBS) followed by another centrifugation to pellet. Adipose stromal cells were stained with human TruStain FcX™ and antibodies to CD11b-PE (BD Biosciences, #557397), CD45-APC (BioLegend, #103112), at 0.25 µg/10^6^ cells. Samples were incubated at 4°C in the dark for 25 min. Following incubation samples were centrifuged to remove unbound antibodies, and the cells were resuspended in PBS containing 5% FBS. GFP, CD45, and CD11b expression were assessed in single stromal cells according to our previously published flow cytometry gating strategy (27).

All cells were sorted at the University of Colorado Cancer Center Flow Cytometry Core facility using a MoFlo XDP cell sorter with Summit 4.3 software. The sheath fluid was IsoFlow, and the sample flow rate was set to a pressure differential of less than 0.4 psi. Sort mode was set to Purify 1. Appropriate signal compensation was set using single color and fluorescent minus one control samples.

### Adipose Tissue Depot Luciferase Assay

At the end of the study, 10–12 weeks posttransplant, mice were euthanized immediately after whole-body imaging. Gonadal, inguinal, and interscapular fat pads along with lower hind limb skeletal muscle (combined gastrocnemius and soleus), liver, and lung were harvested and analyzed for organ-specific luminescence. IVIS imaging data were processed with Living Image 3.0 software. 100 mg of each adipose tissue depot was homogenized, and cytosolic extracts were assayed with the Promega Dual-Luciferase Reporter Assay System (#E1910).

### GFP DNA

Adipocytes from mice that underwent BMT from transgenic mice in which GFP was ubiquitously expressed were isolated by collagenase digestion and centrifugation as described earlier. The freshly isolated adipocytes were immediately processed with the Qiagen DNA Micro kit (#56304). Additional DNA was isolated from GFP transgenic or wild-type animals to be used for a control dilution series of known GFP DNA concentrations. Isolated DNA was quantified on a nano-drop ND1000 spectrophotometer, and subsequent PCR reactions were prepared at a standard concentration of 5 ng genomic DNA per reaction. A housekeeping gene, Gapdh, was simultaneously quantified and used to normalize the GFP expression by ΔCt. The percentage of GFP DNA was calculated from regression analysis of the control dilution series ΔCt values. qPCR reactions were prepared using the Thermo DyNAmo Flash SYBR Green qPCR kit (#F415) according to manufacturer’s directions with primers diluted to a final concentration of 0.5 µM per reaction. qPCR primers were purchased from Integrated DNA Technologies and are as follows (listed as 5′–3′) Gapdh forward: TAC GCA TTA TGC CCG AGG AC, Gapdh reverse: TGT AGG CCA GGT GAT GCA AG, GFP forward: CCA CAT GAA GCA GCA GGA CTT, GFP reverse: GGT GCG CTC CTG GAC GTA. qPCR reactions were run in triplicate on an Applied Biosystems StepOne real-time PCR system.

### Statistical Analysis

Between-group comparisons were evaluated using one-way (body weight and circulating factors) or two-way (depot weight, luciferase, and GFP measurements; group × depot) ANOVA. When indicated by a significant *F*-statistic, *post hoc*-analyses to determine significant mean differences between the groups were conducted with the Bonferroni or Dunnett multiple comparison tests (adjusted *p*-values presented). All results are presented as mean ± SEM unless otherwise noted, and α was set at 0.05. Statistical analysis was completed in GraphPad Prism v7.03.

## Results

### Sex Difference in Accumulation of BM-Derived Adipocytes

**Figure 1:**
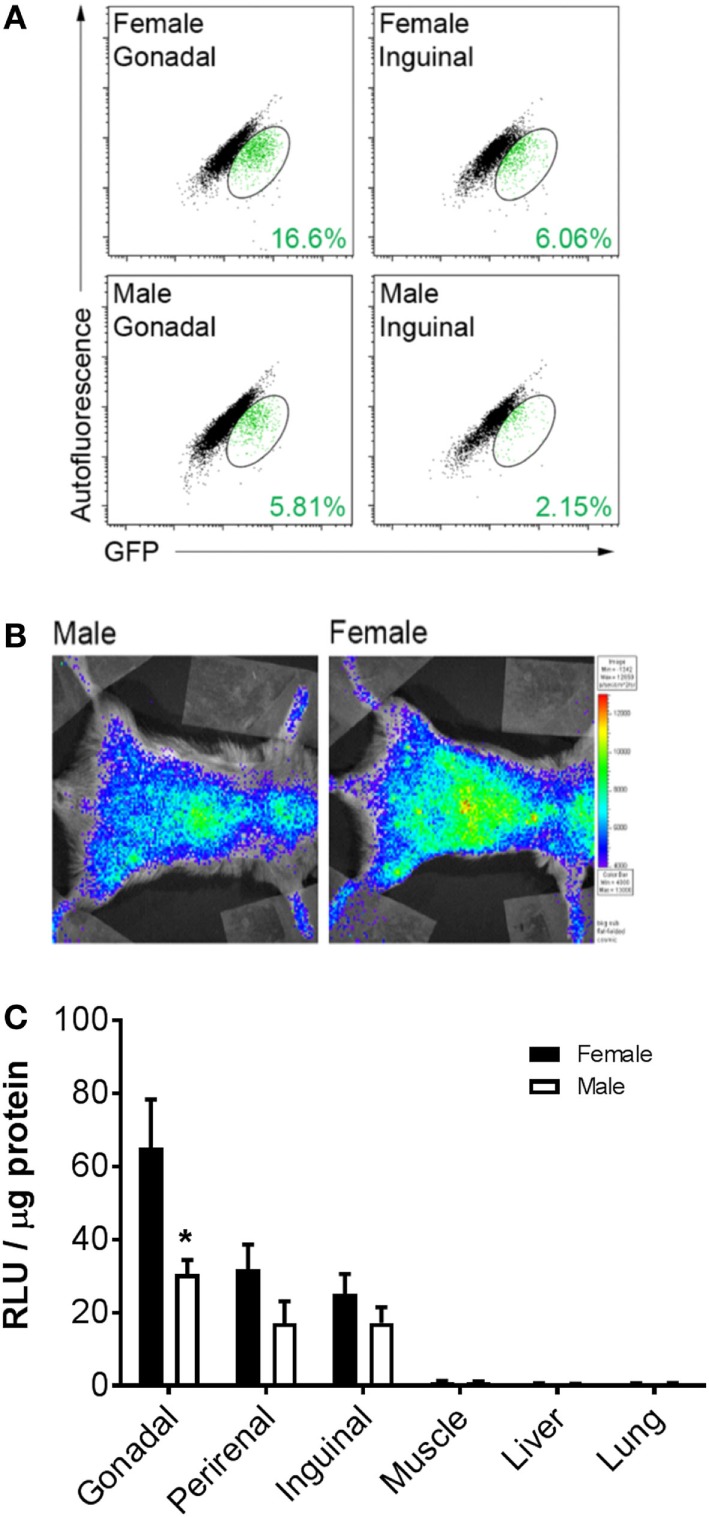
Bone marrow-derived adipocyte (BMDA) production is higher in female rather than age-matched male mice. **(A)** Eight-week-old wild-type male and female mice underwent bone marrow transplantation from sex-matched transgenic mice in which green fluorescent protein (GFP) was ubiquitously expressed. After 12 weeks, gonadal and inguinal fat depots were harvested. Cytosolic GFP expression was assessed in intact adipocytes (LipidTOX Red^POS^ events) containing a single nucleus (dyecycle violet^POS^ events) by flow cytometry. The percentage of BMDAs is denoted in green font in the lower right-hand corner of each scattergram. **(B)** Eight-week-old male and female wild-type mice underwent transplantation with BM from mice in which expression of a luciferase reporter gene was under the control of the adipocyte-specific adiponectin gene promoter (AdipoQ-luciferase donor mice). Twelve weeks posttransplant, whole-body luciferase activity (light emission) was measured in the recipients. Body-wide luciferase activity was consistently higher in female rather than male mice as shown by the representative images. **(C)** Adipose depots (gonadal, perirenal, and inguinal) and non-adipose depots (muscle, liver, and lung) were harvested from male and female AdipoQ-creLSL-Luciferase mice 12 weeks after transplant (*n* = 3 for each sex). Luciferase activity in lysates from each tissue revealed higher luciferase activity in adipose tissue from female than male mice. *p < 0.05 vs female of same depot. Data presented as mean ± SEM.

To determine whether BMDA production is higher in female or male mice, cytosolic GFP expression was measured in intact adipocytes (LipidTOX Red^POS^ events) containing a single nucleus (DyeCycle Violet^POS^ events) by flow cytometry as an indicator that the adipocytes arose from a BM origin. The data show greater production of GFP^POS^ BMDA (green events enclosed in ovals) in both gonadal and inguinal adipose depots of female rather than male mice (Figure 1A). Measurement of whole-body luciferase activity (light emission) in mice that were transplanted with BM from which expression of a luciferase reporter gene was under the control of the adipocyte-specific adiponectin gene promoter demonstrated the same sex difference in production of BMDAs as indicated by greater whole-body light emission in female mice (Figure 1B). Luciferase activity in lysates from individual adipose tissue depots revealed higher luciferase activity in depots from female compared with male mice (main effect of sex *p* < 0.01), although only the difference in the gonadal depot reached statistical significance (*p* = 0.005, Figure 1C). Luciferase activity was essentially absent in non-adipose tissues. Thus, at least in reproductively capable animals, female mice produce more BMDAs than male mice.

### Body and Adipose Tissue Depot Weights

To investigate how ovarian hormones regulate the production of BMDA only female animals were included in all studies from here forward. To generate groups with different ovarian hormone levels we used WT or OVX mice with or without exogenous E_2_. Because ERα is necessary for estrogen to suppress adipogenesis (24), we also utilized whole-body αERKO mice.

**Figure 2:**
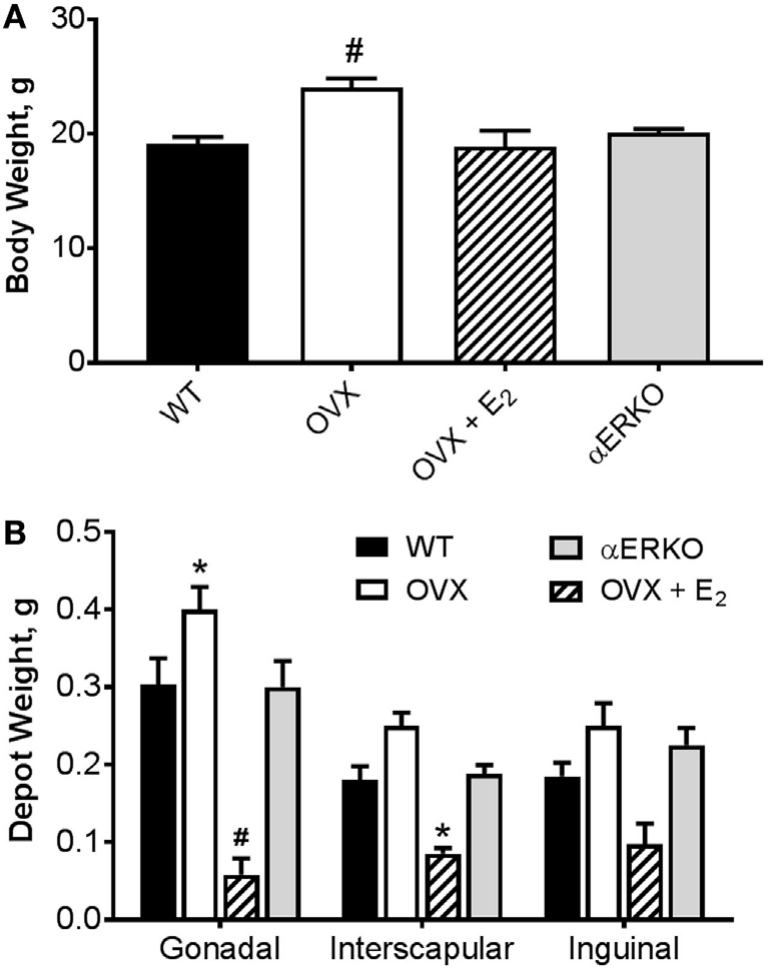
Ovariectomy-induced increases body weight and adipose tissue depot weight are prevented with estradiol replacement. **(A)** Body weight was greater in OVX compared with WT mice. ^#^*p* < 0.001 vs WT [WT *n* = 14, OVX *n* = 7, OVX + E_2_ *n* = 5, estrogen receptor alpha knockout (αERKO) *n* = 6]. **(B)** Gonadal depot weight was higher in OVX and lower in OVX + E_2_ mice compared with WT. Interscapular fat depot weight was lower in OVX + E_2_ mice compared with WT. Inguinal depot weights were not different from WT in any group. *p < 0.05 or ^#^*p* < 0.001 vs WT in same depot (WT *n* = 10, OVX *n* = 6, OVX + E_2_ *n* = 4, αERKO *n* = 5). Data presented as mean ± SEM.

As expected, by 18 weeks of age (8 weeks post-BMT and after 6 weeks of E_2_/P_4_ add-back) there was a significant difference in body weight between the groups (main effect of group, *p* = 0.0002; Figure 2A). OVX mice were heavier than WT (*p* = 0.0001). Body weight was not different from WT in any other group. Similarly, OVX mice had larger, and OVX + E_2_ mice smaller, gonadal fat pads than WT (*p* = 0.02 and *p* = 0.0001, respectively; Figure 2B). Estrogen replacement also resulted in a smaller interscapular fat pad compared with WT (Figure 2B). There were no differences between the groups in the inguinal depot.

### Production of BMDA With Sex Hormone Manipulation

**Figure 3:**
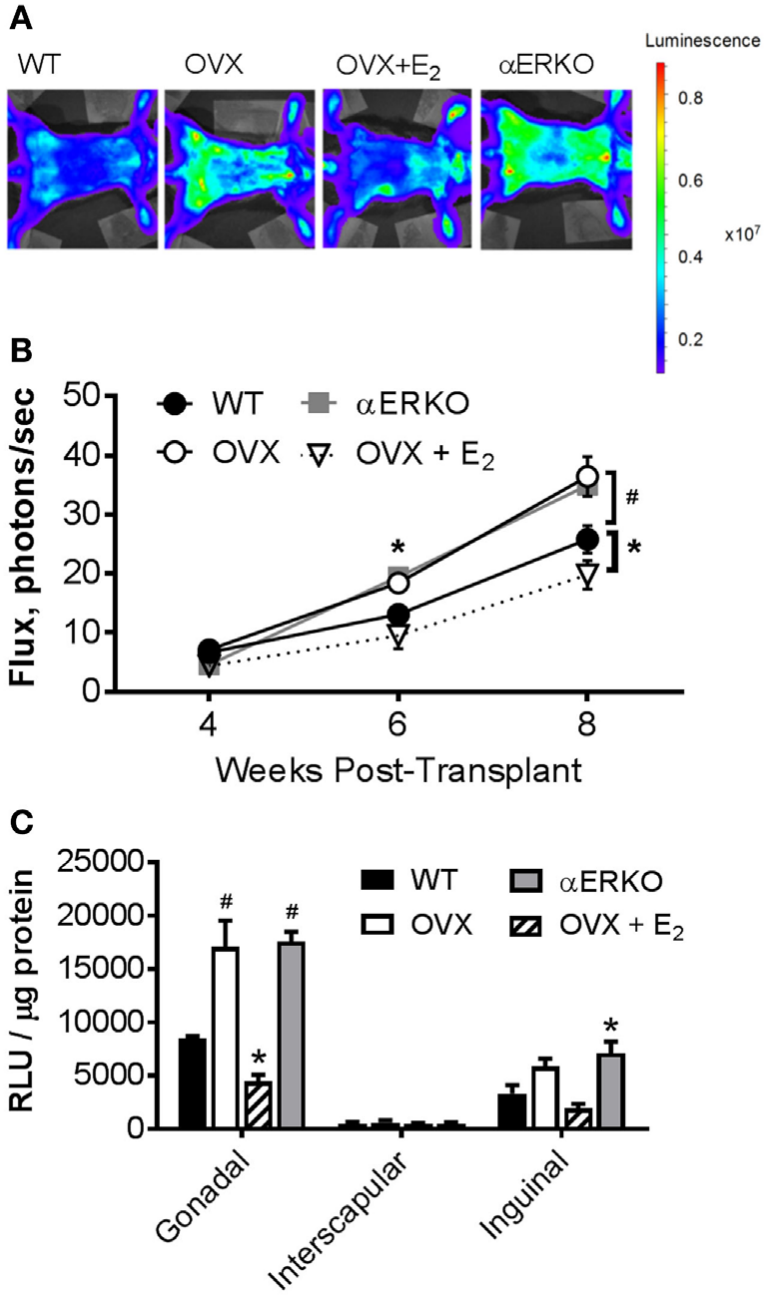
Ovariectomy increases production of bone marrow-derived adipocytes (BMDA) in mice as determined by aP2-cre X LoxP/stop/loxP-Luciferase lineage quantitation. Bone marrow transplantation from donors in which luciferase expression was guided by the fatty acid binding protein 4 (also called adipocyte protein 2 or aP2) gene promoter (aP2-luciferase donor mice) was performed at 8 weeks of age in wild-type recipient mice. Luciferase activity (light emission in the wild-type recipient mice) indicates production of BM-derived adipocytes. **(A)** Representative images of whole-body light emission. **(B)** *In vivo* whole-body light emission measurements were completed 4, 6, and 8 weeks posttransplant. *p < 0.05 and ^#^*p* ≤ 0.001 vs WT at same time point (WT *n* = 4, all other groups *n* = 3). **(C)** Immediately after the last whole-body imaging measurement the mice were euthanized, and gonadal, interscapular and, inguinal fat pads harvested and analyzed for luminescence [expressed as relative light units (RLU)]. *p < 0.05 and ^#^*p* = 0.0001 vs WT in each depot; WT *n* = 4, all other groups *n* = 3. Data presented as mean ± SEM.

We measured light emission at the whole body and adipose tissue depot levels to determine if increases in whole body and fat pad weight could be resultant to the production of new BMDAs. By 6-weeks post-BMT, OVX and αERKO mice had significantly more BMDAs compared with WT (OVX *p* = 0.05, αERKO *p* = 0.003) as indicated by whole-body light emission (Figure 3B). At eight-weeks post-BMT, OVX and αERKO mice still had more BMDAs (*p* = 0.0002 and *p* = 0.0013, respectively), but OVX + E_2_ mice also had fewer (*p* = 0.04), BMDAs compared with WT (Figures 3A,B). At the adipose tissue depot level, on average, luciferase activity was lowest in the interscapular depot (main effect of depot *p* < 0.0001, Figure 3C). Consistent with the whole-body luciferase results, gonadal adipose tissue from OVX and αERKO mice and inguinal adipose tissue from αERKO mice had more BMDAs than WT (all *p* < 0.01). Once again, estrogen replacement completely attenuated, and even decreased, production below WT levels in the gonadal depot (*p* = 0.006 vs WT).

**Figure 4:**
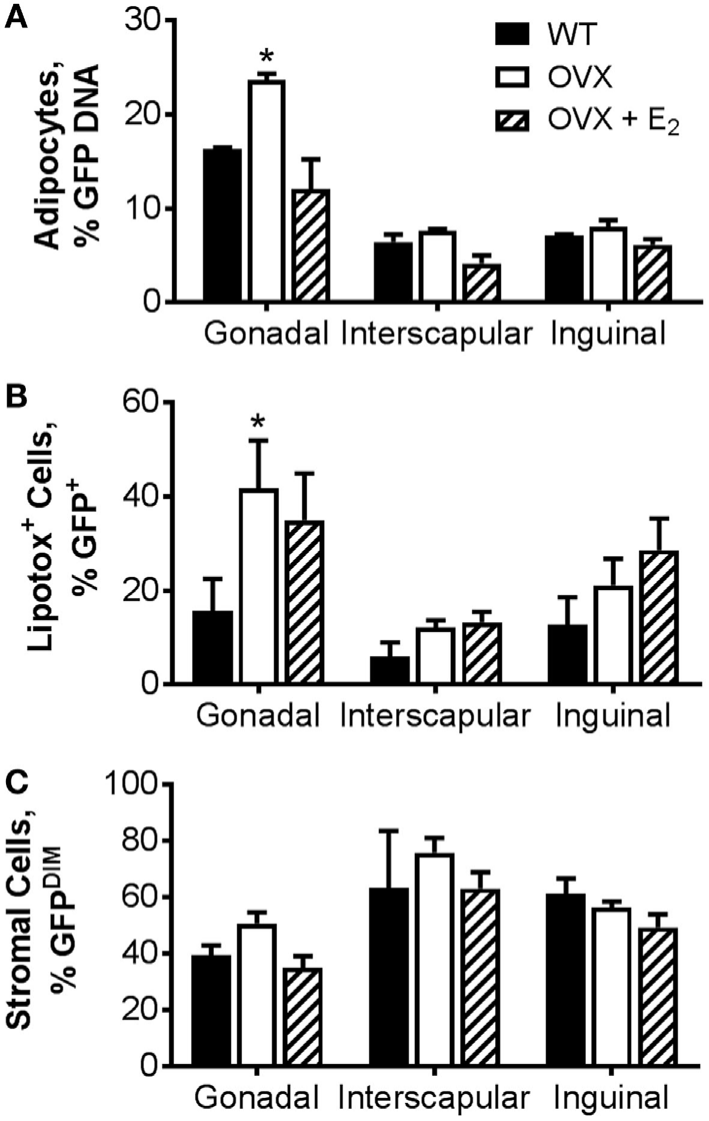
Ovariectomy increases production of bone marrow-derived adipocytes (BMDAs) as determined by green fluorescent protein (GFP) lineage labeling. Eight-week-old WT or OVX mice underwent bone marrow transplantation from transgenic mice in which GFP was ubiquitously expressed. After 2 weeks, a subset of OVX animals was randomized to receive E_2_ replacement (all *n* = 3). After 8 weeks, mice were euthanized, and adipose tissue from gonadal, interscapular, and inguinal fat depots harvested. **(A)** The percent of floated adipocytes cells containing GFP DNA normalized for Gapdh was quantified in floated adipocytes from each depot. **(B)** Percentage of intact adipocytes that were GFP^+^ (BMDAs) as analyzed by flow cytometry. **(C)** Percentage of stromal cells that were GFP^DIM^ (representing bone marrow-derived cells) as analyzed by flow cytometry. *p < 0.05 vs WT in same depot. Data presented as mean ± SEM.

As a complementary measurement to the adipocyte-specific luciferase model, we also utilized a model in which BM from transgenic mice in which GFP was ubiquitously expressed was transplanted into WT or OVX mice, some with E_2_ replacement. In this model, the GFP DNA content in isolated adipocytes from specific adipose tissue depots (Figure 4A) or the percentage of GFP^POS^ adipocytes measured by flow cytometry (Figure 4B) was indicative of BMDA production. As described in detail previously (27), BMDA progenitor cell accumulation was quantified by the percentage of GFP^dim^ cells present in the adipose tissue stroma (Figure 4C). Consistent with the luciferase model, BMDA production was the greatest in the gonadal depot (main effect of depot, *p* < 0.0001 for GFP DNA and *p* = 0.004 for GFP^POS^ adipocytes by flow; Figures 4A,B). Gonadal fat from OVX mice demonstrated the greatest production of BMDAs (*p* = 0.004 vs WT for GFP DNA and *p* = 0.02 for flow), which was once again prevented with estrogen replacement. The sex hormone intervention did not appear to have a significant effect on accumulation of BMDA progenitor accumulation in any depot (Figure 4C).

## Discussion

Here, we demonstrate for the first time that ovarian hormone status in female mice regulates the production of BMDAs. Utilizing multiple transgenic fate mapping models, we observed that surgical depletion of ovarian hormones post-ovariectomy augmented the production of BMDAs in the gonadal depot. Furthermore, estradiol replacement consistently attenuated the accelerated production of BMDAs. In addition, genetic knockdown of ERα also enhanced BMDA production in both the gonadal and inguinal depots. Taken together, our data suggest that estradiol regulates BMDA production.

The mechanisms underlying the shift toward preferential abdominal adiposity with decreased circulating ovarian hormones in both women and female mice are not completely understood. Importantly, increased central adiposity is associated with elevated risk for cardiovascular disease (28), a leading cause of death in postmenopausal women. Thus, continued pursuit of uncovering the mechanisms underlying this phenomenon in women over the menopausal transition is critical in maintaining women’s health across the lifespan.

Central adiposity may be imposing detrimental health effects not only through mechanisms associated with increased adipose tissue volume but also changes in its adipocyte composition. Although non-adipocyte cell accumulation, local blood flow and neural innervation are important determinants of the local adipose tissue microenvironment, inherent characteristics of the constituent adipocytes differ by depot. Importantly, these depot-specific characteristics are retained even when the cells are studied *in vitro* (e.g., adiponectin secretion, insulin action, and signaling) (8–10, 29). Recently, alternatives to the traditional adipose tissue resident mesenchymal lineage origin for adipocytes have been proposed (e.g., BM progenitors or BMDCs) (30). Thus, adipocytes arising from varying lineages of multipotent mesenchymal progenitors (9, 11, 31, 32) and hematopoietic progenitors (15) may contribute to differences in adipocyte characteristics and phenotypes both between and within adipose tissue depots. However, in adults, the initiating or limiting factors for the production of new adipocytes from varying lineages remain uncertain. Better understanding of these mechanisms may uncover new targets for novel health and weight management strategies.

### Sex Differences

The BMDA is a novel “alternative lineage” adipocyte, a likely candidate in the regulation of adipose tissue heterogeneity. We previously observed a sex difference in the production of BMDAs in male and female mice, confirmed herein, with female mice producing more BMDAs compared with males (30). Although observed in all major fat depots, the highest production of BMDAs is evident in gonadal fat, considered a visceral fat depot in mice. Global gene expression analysis reveals a detrimental metabolic phenotype characterized by inflammatory and mitochondrial related genes divergently expressed between conventional white and BMDAs (15), further supporting the contribution to depot heterogeneity.

The greater production of BMDAs in young female compared with male mice appears counterintuitive. However, females have higher total and percent body fat compared with males. Thus, females may have more BMDAs throughout life resultant to their greater relative fat mass. In addition, the rise in plasma estradiol at puberty and the decline with menopause are both associated with increased fat mass in women. A similar dual-phase relationship may exist with estradiol regulation of BMDA production in female mice. Whether the sex difference observed in mice translates to humans is unknown.

### Alterations in Cellular Composition

Animal models are ideal for exploring the relationship between E_2_ and the adipocyte. Alterations in the production of new adipocytes (adipogenesis) as well as adipocyte size occur with the loss of circulating ovarian hormones in mice. For example, ovariectomy leads to an increase in adipocyte size, along with increases in overall body fat and visceral adiposity, which is fully preventable by E_2_ administration (20). Similarly, the aromatase knockout mouse, which is unable to synthesize endogenous estrogens, has increased body weight and visceral adiposity compared with WT animals (33, 34). E_2_ replacement prevents this increase in body weight and central adiposity and decreases adipocyte size (33, 34). Finally, αERKO mice exhibit increased adiposity, particularly in the visceral depots accompanied by increases in adipocyte volume and number (21). Importantly, exogenous estrogen therapy in these mice is not effective at reducing fat mass (23). Thus, it is clear that estrogen status, and signaling through ERα in particular, has an important effect on adipose tissue volume and cellularity. However, if the “type” of adipocytes primarily produced or altered in the estrogen-deficient state are the same as those in the estrogen replete state is unknown.

Unlike previous studies, compared with WT we did not observe elevated fat mass in our αERKO mice. This could be due to the fact that irradiated animals and/or animals maintained at Aurora, CO, USA, altitude (1,600 m) eat less, gain less weight, and exhibit decreased adiposity compared with unirradiated animals maintained at lower altitudes (12). On the other hand, we did observe the expected OVX-induced increase in body weight and adiposity. This difference between models may have been a result of the relatively short timeline (8–10 weeks after transplant) over which the study was conducted. Within 15 days, OVX mice already have a higher body weight compared with sham controls (35), while differences in body weight in αERKO are not reported until 12–16 weeks (21, 36–39). Importantly, our results suggest that weight gain is not a prerequisite for the production of BMDAs, at least resultant to disrupted estrogen signaling. Whether a preferential increase in the production of BMDAs compared with conventional lineage adipocytes is responsible for the observed OVX-induced weight gain cannot be determined from the methods utilized in this study. However, studies investigating the turnover rate of both lineages of adipocytes are critical to answer this question.

Both Vieira Potter et al. (40) and Rogers et al. (19) observed increased macrophage infiltration and activation in gonadal adipose tissue as early as 12 weeks post-OVX. Notably, BMDA appear to develop *via* a novel transdifferentiation of adipose tissue macrophages (27), and we consistently observe their highest production in the gonadal fat depots (15, 18). However, we did not observe a difference in the percentage of BMDA progenitors (i.e., GFP^DIM^ cells) in adipose stroma between WT and the OVX, OVX + E_2_ or αERKO groups. Similar results were obtained in mice treated with high fat diet or rosiglitazone, both of which exhibited more mature BMDAs without altered accumulation BM-derived progenitors in the SVF (12). Of note, we did not quantify the proportion of different SVF cell types (i.e., macrophage, T and B lymphocytes, neutrophils, eosinophils, dendritic cells, endothelial cells, fibroblasts, and mesenchymal stem cells) or the activation state of resident macrophages. Either of those measurements could reveal alterations at the SVF level that were not detected with our generalized GFP quantification of a heterogeneous population of stromal cells.

Our findings suggest that changes in ovarian hormone status/signaling result in alterations in the cellular composition of adipose tissue that may not contribute to alterations in the size of the adipose tissue depot. Notably, previous research indicates that obesity *per se* is not required for the development of menopause-associated metabolic disturbances (40, 41). Thus, future studies are warranted to investigate the relationship between weight gain/energy balance, rate of production of BMDAs and declines in metabolic health after OVX in female mice.

### Role for Estradiol in BMDA Production

Our results consistently demonstrated that estradiol replacement successfully attenuates the production of BMDAs as well as the OVX-induced increase in body weight. It appears as though E_2_ signaling through ERα is one mechanism by which estrogen may regulate the production of BMDAs. These studies complement existing evidence that estrogen has potentially beneficial effects on the adipose tissue through ERα signaling. ERα has been identified has having important roles in maintaining not only adipocyte size and number but also adipose tissue inflammation and fibrosis, as demonstrated in adipose tissue knockout and gonadal depot-specific ERα knockdown (42). Interestingly, mouse models specifically knocking out ERα in hematopoietic or myeloid lineage cells, or transplanting ERα^−/−^ BM into LDL Receptor knockout mice, all result in increased adipose tissue mass (43). The additional adipose tissue in these mice is accompanied by increased adipocyte size, number of crown like structures, chemokine expression, and immune cell infiltration and inflammation in the gonadal adipose tissue. Increased accumulation of BMDCs in the adipose tissue of these mice, along with their observed insulin resistance, demonstrates the importance of myeloid cell ERα expression in the maintenance of adipose tissue homeostasis (43).

It is also possible that the removal of ERα in our model resulted in increased BMDA accumulation through greater ERβ signaling. Because of disrupted negative feedback regulation, circulating estrogen levels can be higher in αERKO than WT (22, 44), and the increase in fat mass observed in αERKO mice is attenuated with OVX, suggesting that estrogen signaling through ERβ plays at least some part in the elevated fat mass evident in αERKO mice (45). Because we did not perform OVX in our αERKO mice we cannot rule out a role for ERβ in the increased production of BMDAs. However, our consistent results between the OVX (low circulating estrogens and intact ERα) and αERKO (high circulating estrogens and low ERα) models suggest reduced signaling through ERα does play a role. Future studies designed specifically to isolate the roles of ERα, ERβ, and the G protein-coupled estrogen receptor (GPER or GPR30) are critical in the complete understanding of the role of E_2_ in production of BMDAs.

### Limitations

While OVX is often used as a rodent model of menopause, the surgical intervention results in a rapid decline in ovarian hormones and eliminates the perimenopausal period when women experience fluctuations in hormone levels and irregular menstrual cycles. Although a very useful model for basic mechanistic studies, future studies utilizing the chemical 4-vinylcyclohexene diepoxide to mimic a more prolonged variable menopausal transition will provide a translation to the perimenopausal period. Furthermore, the mice were ovariectomized at 8 weeks of age, which, although not uncommon, does not recapitulate the older age at which women would usually undergo natural (or even surgical) menopause. In addition, OVX was conducted before the BMT. It is possible that OVX occurring at a later age, or after the BMT, would alter our findings.

Although the effects of OVX and E_2_ add-back on BMDA production were only statistically significant in the gonadal depot, the inguinal depot tended to respond similarly. It is possible that the lower overall BMDA production in the inguinal depot as well as the small sample size limited our statistical power to detect significant differences between the groups in this depot. Continuing to study the region-specific production of BMDAs will be important in future studies.

The E_2_ add-back regimen utilized 0.05 mg 60-day constant release pellets. This was expected to maintain circulating estrogen levels within the physiological range. However, a limitation of this study is that we do not have the samples available to measure circulating E_2_ in our mice to confirm the actual values. Our observation that gonadal adipose tissue weight was lower in the OVX + E_2_ compared with WT suggests the E_2_ replacement could have been supraphysiologic. However, whole body or inguinal adipose depot weights were not statistically different between the WT and OVX + E_2_ groups. Furthermore, previous literature reports that higher dose E_2_ pellets (e.g., 0.25 or 0.1 mg) result in circulating E_2_ levels well within physiological range (<117–734 pmol/l) (46, 47) therefore it is unlikely, although not impossible, that our low dose resulted in supraphysiological replacement. In addition, whether a constant release single hormone add-back regimen leads to different results than a more physiological cyclical combination hormone replacement is uncertain.

The whole-body αERKO mice used in this study carried the *Esr1^tm1Ksk^* allele. These mice express a low level of a truncated form of ERα due to alternate splicing of the transcript resulting in a small amount of estrogen binding (~3%) in the uterine tissues of the homozygote animals as compared with wild-type controls. Importantly, no E_2_ binding was detected in other tissues of this strain of animal (e.g., brain, kidney, and liver) (The Jackson Lab, Stock No. 004744; https://www.jax.org/strain/004744). Recently, Fatima and colleagues reported estrogen receptor 1 (*Esr1* or ERα) mRNA expression is significantly lower in gonadal and inguinal adipose tissue from this same strain of ERKO mice compared with C57BL WT controls (48). However, because measurement of BMDA production required use of the entire fat pad, we did not confirm the lower the *Esr1* gene expression in the adipose tissue of the mice studied here.

## Conclusion

Our results suggest that, in female mice, the loss of ovarian hormone results in increased production of BMDAs. In particular, the loss of estradiol, possibly through ERα appears to be important in regulating the production of *de novo* BMDAs. Thus, a shift in the adipocyte composition of adipose tissue depots toward more BMDA production with the loss of ovarian hormones in females could be related to the increased metabolic disease risk observed in this population. Studies to investigate the metabolic phenotype and physiological impact of the production of BMDAs are needed before a direct mechanistic link can be confirmed.

## Data Availability Statement

The raw data supporting the conclusions of this manuscript will be made available by the authors, without undue reservation, to any qualified researcher.

## Ethics Statement

All animal procedures were performed in an AAALAC-accredited facility in accordance with the Guide for the Care and Use of Laboratory Animals (25) and approved by the University of Colorado Denver Institutional Animal Care and Use Committee.

## Author Contributions

DK, SM, and WK contributed to conception and design of the study; TS and DK performed data collection; KG and DK performed data analysis; KG, DK, SM, and WK performed data interpretation; KG wrote the first draft of the manuscript; KG, TS, and DK wrote sections of the manuscript. All the authors contributed to manuscript revision, read and approved the submitted version.

## Conflict of Interest Statement

The authors declare that the research was conducted in the absence of any commercial or financial relationships that could be construed as a potential conflict of interest. The reviewer KM and handling Editor declared their shared affiliation.
